# WHAT MOTIVATES PARTICIPATION IN ALZHEIMER’S PREVENTION RESEARCH: A MIXED-METHODS STUDY

**DOI:** 10.1093/geroni/igac059.2779

**Published:** 2022-12-20

**Authors:** Lizbeth Vera Murillo, Maria Vander Meulen, Monique Villamor, Angel Collie, Sarah Cline, Jody Nicholson, Jerri Edwards

**Affiliations:** University of North Florida, Jacksonville, Florida, United States; University of North Florida, Jacksonville, Florida, United States; University of North Florida, Jacksonville, Florida, United States; University of North Florida, Jacksonville, Florida, United States; University of North Florida, Jacksonville, Florida, United States; University of North Florida, Jacksonville, Florida, United States; University of South Florida, Tampa, Florida, United States

## Abstract

6.5 million Americans aged 65 and older are currently living with Alzheimer’s disease or related dementias (ADRD; Alzheimer’s Association, 2022). By 2060, it is estimated that this number will increase to 13.9 million (Matthews et al., 2018). Therefore, it is imperative to gain insight into participants’ personal motivations and expectations of research to advance community participation. The Preventing Alzheimer’s with Cognitive Training (PACT) study is a National Institute of Health, National Institute on Aging-funded, multi-site clinical trial examining the prevention of mild cognitive impairment and ADRD through computer-based cognitive training. Across 5 locations, data were collected from 2,360 cognitively normal participants (M=73.03 years, range=65–97, SD=5.04). The current project explores individuals’ motivations and expectations of cognitive training (CT) utilizing a mixed-method approach by coding qualitative open-ended questions about motivation to participate and comparing how motivational themes aligned with expectations about CT from the Expectations Assessment Scale (EAS; Rabipour et al., 2018). Six themes for participant motivation emerged: direct experience with the disease (26.9%), concern about brain health and aging (23.9%), general personal interest (17.9%), general interest in research (20.1%), referral to the study (5.8%), and altruism (5.4%). After completing the initial training session, motivation themes did not differentiate satisfaction with (p=.06) or perceived success of (p=.11) the CT program. Understanding participants’ motivations can further expand and optimize recruitment and retention strategies in AD prevention research. Future research will focus on how these themes influence adherence and retention and relate to participant demographic characteristics (i.e., education, gender, race, and ethnicity).

